# Molecular epidemiological and virological study of dengue virus infections in Guangzhou, China, during 2001–2010

**DOI:** 10.1186/1743-422X-10-4

**Published:** 2013-01-02

**Authors:** Liyun Jiang, Xinwei Wu, Yejian Wu, Zhijun Bai, Qinglong Jing, Lei Luo, Zhiqiang Dong, Zhicong Yang, Yang Xu, Yimin Cao, Biao Di, Yulin Wang, Ming Wang

**Affiliations:** 1 Guangzhou Center for Disease Control and Prevention, 1 Qide Road, Guangzhou, Guangdong 510440, China

**Keywords:** Dengue virus, Infection, Molecular biology, Phylogenetic analysis, South China

## Abstract

**Background:**

Dengue virus (DENV) infection is the most prevalent arthropod-borne viral infection in tropical and subtropical regions worldwide. Guangzhou has the ideal environment for DENV transmission and DENV epidemics have been reported in this region for more than 30 years.

**Methods:**

Information for DENV infection cases in Guangzhou from 2001 to 2010 were collected and analyzed. The DENV strains were cultured and isolated from patients’ sera. Viral RNA was extracted from cell culture supernatants. cDNA was synthesized by reverse transcription PCR. Phylogenetic trees of four DENV serotypes were constructed respectively.

**Results:**

In total, 2478 DENV infection cases were reported; 2143 of these (86.43%) occurred during 3 months of the year: August, September and October. Of these, 2398 were local cases (96.77%) and 80 were imported cases (3.23%). Among the imported cases, 69 (86.25%) were from Southeast Asian countries. From the 90 isolated strains, 66.67%, 3.33%, 14.44%, and 15.56% belonged to DENV serotypes 1, 2, 3, and 4, respectively. DENV-1 was predominant in most of the years, including during 2 outbreaks in 2002 and 2006; however, none of the strains or genotypes identified in this study were found to be predominant. Interestingly, DENV strains from different years had different origins. Moreover, the strains from each year belonged to different serotypes and/or genotypes.

**Conclusions:**

Southeast Asia countries were found to be the possible source of DENV in Guangzhou. These findings suggest that there is increasing diversity in DENV strains in Guangzhou, which could increase the risk of DENV outbreaks in the near future.

## Background

Dengue virus (DENV) is a member of the genus *Flavivirus*, family *Flaviviridae*. It is an enveloped virus with an 11-kb positive sense single-stranded RNA genome. The genome encodes a single open reading frame and can be translated into 3 structural proteins, that is, the core (C), premembrane/membrane (prM/M), and envelope (E) proteins, and 7 non-structural proteins, that is, NS1, NS2a, NS2b, NS3, NS4a, NS4b, and NS5. DENV can be divided into 4 serotypes and several genotypes according to the sequence of the E gene. DENV infection can cause differential syndromes ranging from a severe flu-like illness called dengue fever (DF) to lethal complications like dengue hemorrhagic fever (DHF) and dengue shock syndrome (DSS) [1,2]. Infection with any of the 4 serotypes can cause extremely severe manifestations. Moreover, natural infection with any of the serotypes can only provide long-term homotypic immunity, which leads to a higher risk for DHF/DSS during secondary infections with a heterogenous serotype [3,4].

DENV infection has been the most prevalent arthropod-borne viral infection in tropical and subtropical regions worldwide. There are 2 competent vectors of DENV, *Aedes aegypti* and *Ae*. *albopictus*. According to World Health Organization (WHO) estimates, over 100 countries and approximately 40% of the world’s population (nearly 2.5 billion people) are threatened by DENV [5,6]. Currently, approximately 100 million DENV infections occur worldwide every year, and the WHO has classified dengue as a major international public health concern. Globalization has also increased the spread of viruses and mosquito vectors; hence, epidemics and outbreaks have occurred with increased frequency in recent years [7,8]. The diversity of the virus is also increasing since multiple DENV serotypes can co-circulate in the same location within a short time. Evolution, dispersal, and replacement of serotypes and genotypes could also take place during a continuous period under selection pressure [9-13].

Since the first documented DENV infection in Foshan in 1978, DENV has spread in China for more than 30 years, mostly in the south-east coast of China in regions such as the Guangdong, Shanghai, Jiangsu, Zhejiang, Hainan, Guangxi, and Fujian provinces [14]. Guangzhou, which is one of the biggest cities in South China, has a high incidence of DENV infections and in recent decades, DENV infection cases have been recorded in Guangzhou almost every year. The cases include both imported and indigenous cases, and all 4 DENV serotypes have been involved. In this study, epidemiological information pertaining to DENV infection cases that occurred between 2001 and 2010 in Guangzhou was reviewed. DENV strains from DF patients were isolated, and phylogenetic analysis was performed based on the E gene sequences. By combining epidemiological and virus serotype data from DENV cases occurring in Guangzhou between 2001 and 2010, we provide further insights into the transmission patterns of DENV in Guangzhou.

## Results

### Epidemiological characteristics of DENV in Guangzhou from 2001 to 2010

**Figure 1:**
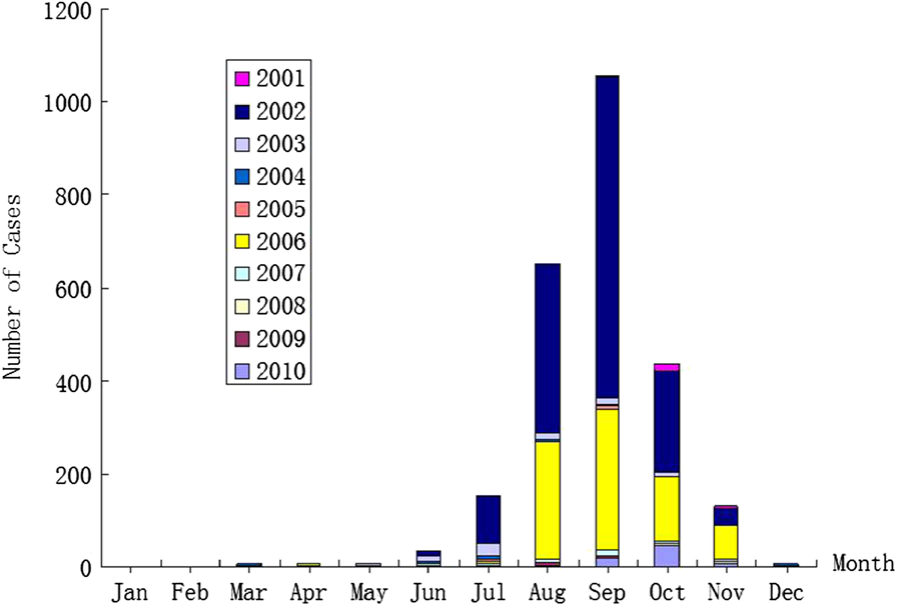
Monthly distribution of reported DENV infection cases in Guangzhou between 2001 and 2010.

Information for all DENV infection cases was collected by the Guangzhou Center for Disease Control and Prevention (Guangzhou CDC). In total, 2478 DENV infection cases were reported; both laboratory and clinically confirmed cases were covered. All the infected patients had DF symptoms; however, no deaths or DHF/DSS cases were found. DENV infections were detected every year in Guangzhou from 2001 to 2010. Figure 1 shows the monthly distribution of reported DENV infection cases; 86.43% (2143 cases) of them occurred during 3 months of the year: August, September, and October.

There were 2398 local cases (96.77%) and 80 imported cases (3.23%) (Table 1). Among the imported cases, 69 (86.25%) came from Southeast Asian countries, including Cambodia, Indonesia, Malaysia, Myanmar, Philippines, Singapore, Thailand, Vietnam, Bangladesh, and India. In addition, 5 (6.25%) were imported from Africa (Niger, Senegal, South Africa, Tanzania, and Sudan), 2 (2.50%) from the Middle East (Saudi Arabia and Yemen), and 1 (1.25%) from America (Dominican Republic). The original source of 3 (3.75%) imported cases could not be identified. The imported cases outnumbered the local cases in 2005, 2008, and 2009. Two major outbreaks occurred in 2002 and 2006.

**Table 1:** Summary of reported DENV infection cases in Guangzhou in 2001–2010

**Year**	**2001**	**2002**	**2003**	**2004**	**2005**	**2006**	**2007**	**2008**	**2009**	**2010**	**Total cases**
Total cases	19	1423	78	21	12	779	35	12	18	81	2478
Local cases	18	1419	76	15	1	771	22	4	6	66	2398
Imported cases	1	4	2	6	11	8	13	8	12	15	80
Country of origin											
Bangladesh	0	0	0	0	1	0	1	0	0	0	2
Cambodia	0	0	0	2	1	4	4	0	0	1	12
Dominican Republic	0	0	0	0	0	0	1	0	0	0	1
India	0	0	0	1	2	0	0	0	0	1	4
Indonesia	1	1	0	2	2	2	1	1	2	2	14
Malaysia	0	0	0	0	1	0	2	2	1	1	7
Myanmar	0	0	0	0	0	0	1	0	0	0	1
Nigeria	0	0	0	0	0	0	0	0	0	1	1
Philippines	0	0	0	0	0	0	0	0	2	0	2
Saudi Arabia	0	0	0	0	0	0	0	0	1	0	1
Senegal	0	0	0	0	0	0	0	0	1	0	1
Singapore	0	0	1	1	3	1	0	0	0	0	6
South Africa	0	0	0	0	0	0	0	1	0	0	1
Sudan	0	0	0	0	0	0	0	0	0	1	1
Tanzania	0	0	0	0	0	0	0	0	0	1	1
Thailand	0	1	0	0	1	0	3	0	2	3	10
Vietnam	0	0	0	0	0	1	0	3	3	4	11
Yemen	0	0	1	0	0	0	0	0	0	0	1
Unknown	0	2	0	0	0	0	0	1	0	0	3

### DENV strains in Guangzhou from 2001 to 2010

We isolated 90 strains from the serum samples of patients by using C6/36 cell line. The full-length DENV E gene sequences were then analyzed and compared. The sequences from 76 strains were deposited in GenBank with the following accession numbers: JN009085-99, HM466962-8, JQ002658, and JQ277834-86. The remaining 14 sequences were found to be identical sequences and were therefore not deposited. All the sequences were named using 2 numbers, one indicating the year and the other indicating the sample number. For example, 07–5668 means the strain was isolated in 2007 and the original sample number is 5668. The reference strain sequences were downloaded from Genbank and named with the serotype, country, separation year, and accession number. In total, 10 of the isolated strains came from imported cases (Table 2), whilst the remaining 80 strains originated locally.

**Table 2:** Serotypes and the countries of origin of the imported strains in phylogenetic trees

**Strain number**	**Serotype**	**Country of origin**
05-226	1	Singapore
05-464	2	Indonesia
07-5668	1	Thailand
07-5757	1	Malaysia
08-7849	1	Vietnam
09-1081	3	Vietnam
09-9104	1	Saudi Arabia
09-9236	1	Saudi Arabia
09-11562	1	Thailand
09-13105	3	Thailand

Among the 90 strains, 66.67%, 3.33%, 14.44%, and 15.56% belonged to DENV serotypes 1, 2, 3, and 4, respectively. Table 3 shows the different serotypes for each year. Although DENV-1 was predominant in most years of the decade, DENV-2, DENV-3, and DENV-4 were occasionally isolated in 2005 and 2009. In 2010, all 4 DENV serotypes appeared were detected.

**Table 3:** Serotype distributions of sequenced DENV strains in 2001–1010

**Year**	**DENV-1**	**DENV-2**	**DENV-3**	**DENV-4**	**Total**
2001	1	0	0	0	1
2002	11	0	0	0	11
2003	1	0	0	0	1
2004	1	0	0	0	1
2005	2	1	0	0	3
2006	27	0	0	0	27
2007	7	0	0	0	7
2008	1	0	0	0	1
2009	3	0	7	0	10
2010	6	2	6	14	28
Total	60	3	13	14	90

### Phylogenetic analysis of DENV-1

**Figure 2:**
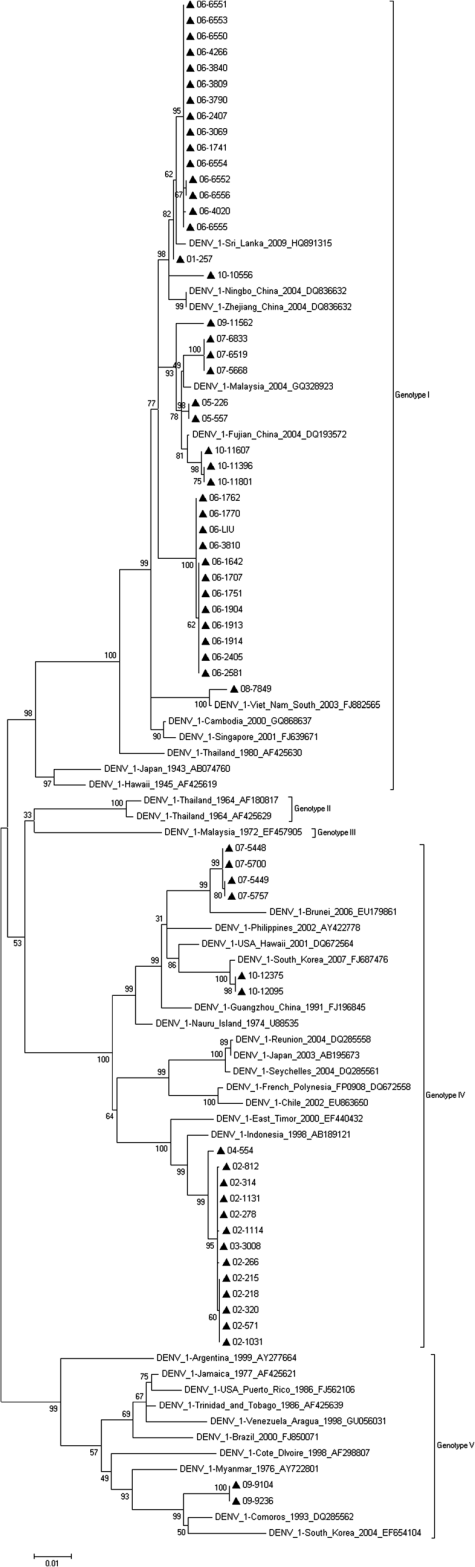
**Phylogenetic tree of the DENV-1 E gene isolated in Guangzhou between 2001 and 2010. **Sixty sequences from isolated strains and 38 reference sequences from GenBank were aligned using ClustalW. Phylogenetic trees were constructed with the maximum parsimony and maximum likelihood methods with Kimura 2-parameter corrections of multiple substitutions. Guangzhou isolates are indicated with a black triangle.

Sequences of the E gene from the 60 DENV-1 isolates were aligned using ClustalW [15] and compared with 38 reference sequences in GenBank. Figure 2 shows the phylogenetic tree of the sequences using the maximum likelihood analysis and indicates that DENV-1 in Guangzhou falls into 3 genotypes. All strains from 2002, 2003, and 2004 clustered in the genotype IV, and all strains from 2001, 2005, 2006, and 2008 clustered in the genotype I. Strains from 2007 and 2010 separated into both genotypes. One strain from 2009 clustered in the genotype I, whilst the other 2 strains clustered in the genotype V.

### Phylogenetic analysis of DENV-2

**Figure 3:**
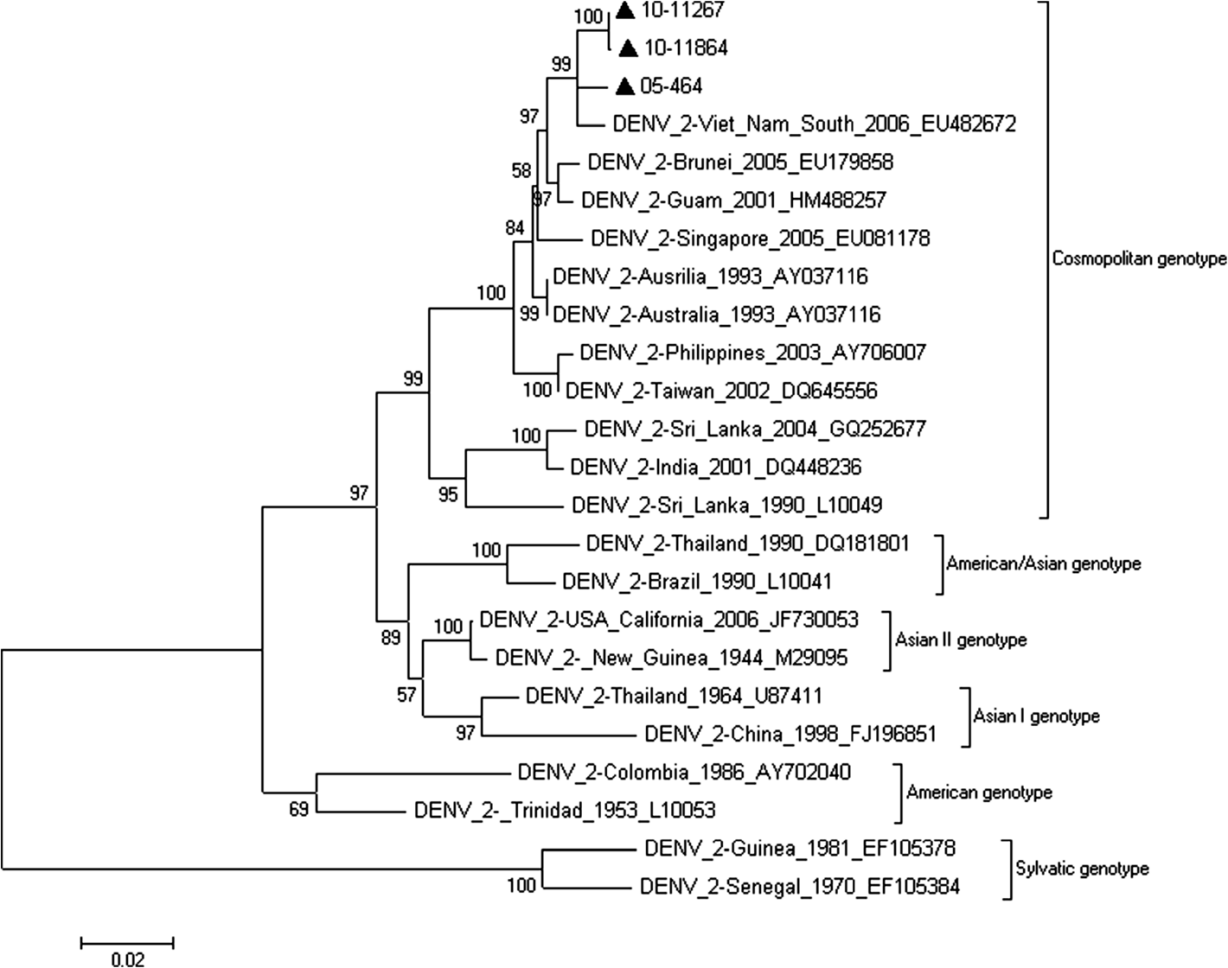
**Phylogenetic tree of the DENV-2 E gene isolated in Guangzhou between 2001 and 2010. **Three sequences from isolated strains and 20 reference sequences from GenBank were aligned using ClustalW. Phylogenetic trees were constructed with the maximum parsimony and maximum likelihood methods with Kimura 2-parameter corrections of multiple substitutions. Guangzhou isolates are indicated with a black triangle.

Only 1 strain of DENV-2 was isolated in 2005, whereas 2 strains were isolated in 2010. Three E gene sequences of DENV-2 were aligned using ClustalW and analyzed with 20 reference sequences from GenBank. Figure 3 shows the phylogenetic tree of the 23 sequences with maximum likelihood analysis. All 3 DENV-2 strains were found to belong to the Cosmopolitan genotype.

### Phylogenetic analysis of DENV-3

**Figure 4:**
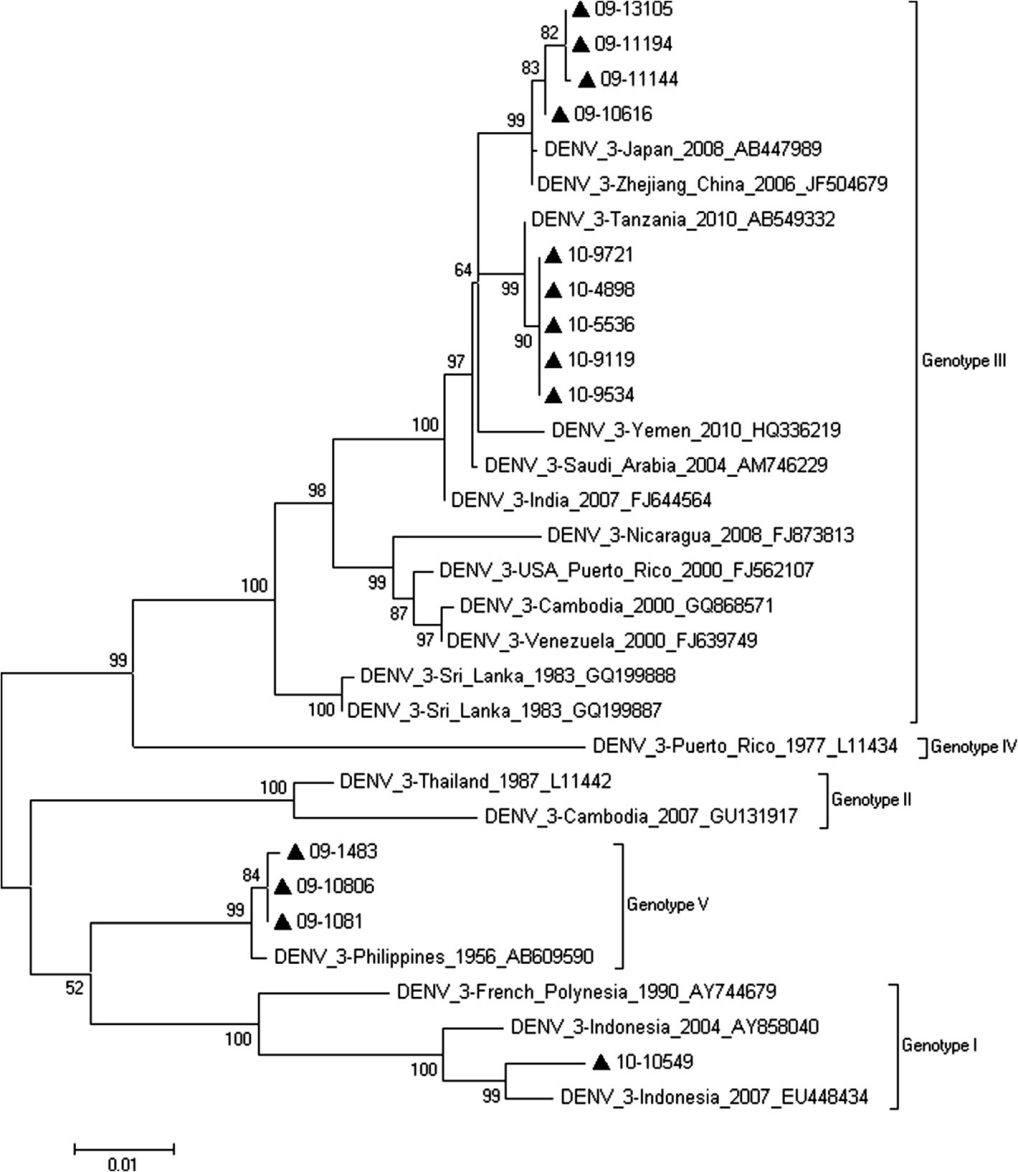
**Phylogenetic tree of the DENV-3 E gene isolated in Guangzhou between 2001 and 2010. **Thirteen sequences from isolated strains and 22 reference sequences from GenBank were aligned in ClustalW. Phylogenetic trees were constructed with the maximum parsimony and maximum likelihood methods with Kimura 2-parameter corrections of multiple substitutions. Guangzhou isolates are indicated with a black triangle.

Thirteen strains of DENV-3 were isolated between 2001 and 2010, 7 in 2009, and 6 in 2010. The strains were aligned using ClustalW and analyzed with 22 reference sequences from GenBank. Figure 4 shows the phylogenetic tree of the 35 sequences with maximum likelihood analysis. The 13 DENV-3 strains consisted of genotype III (9) and genotype V (4).

### Phylogenetic analysis of DENV-4

**Figure 5:**
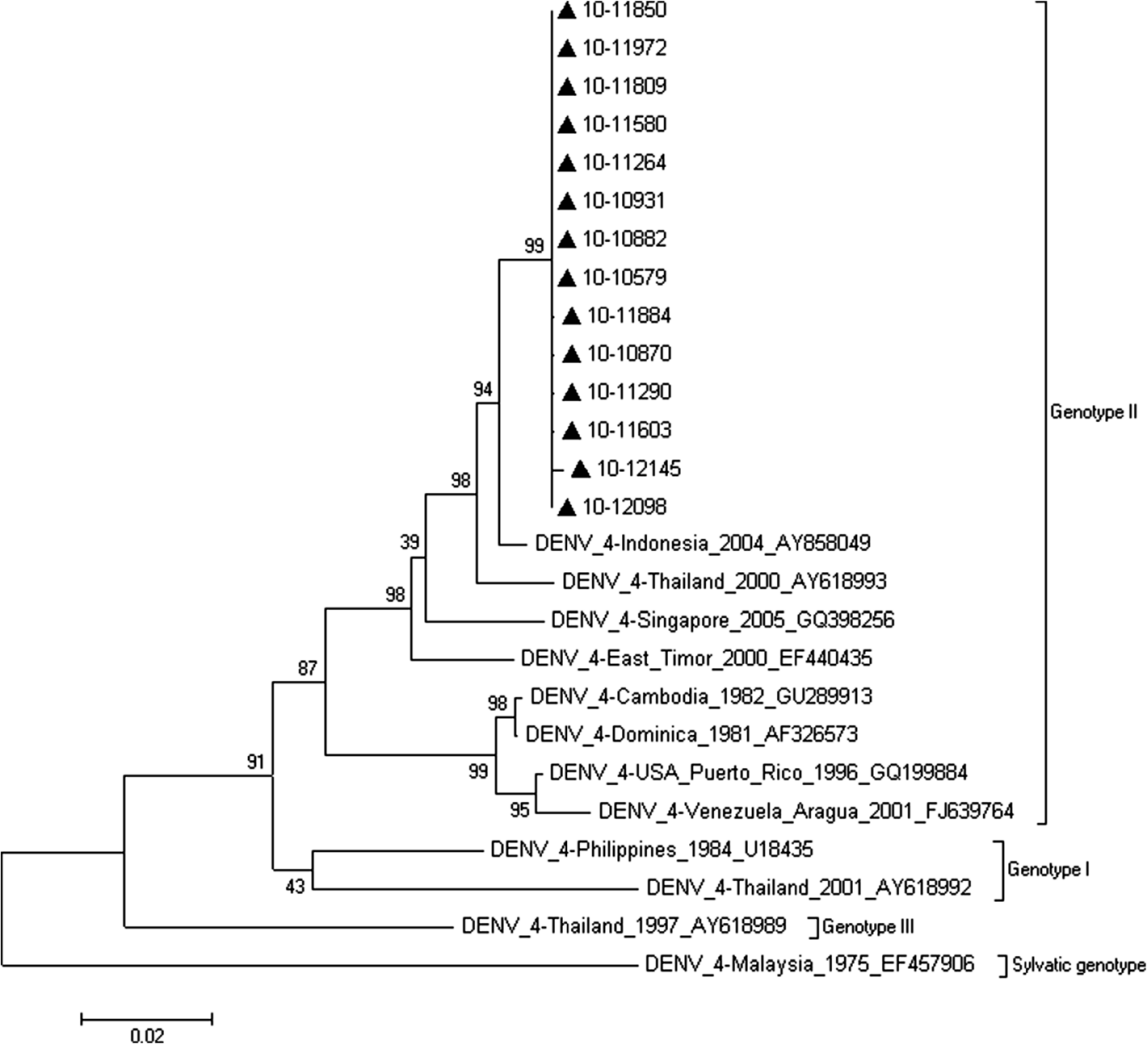
**Phylogenetic tree of the DENV-4 E gene isolated in Guangzhou between 2001 and 2010. **Fourteen sequences from isolated strains and 11 reference sequences from GenBank were aligned using ClustalW. Phylogenetic trees were constructed with the maximum parsimony and maximum likelihood methods with Kimura 2-parameter corrections of multiple substitutions. Guangzhou isolates are indicated with a black triangle.

All 14 DENV-4 strains were isolated in 2010. They were aligned using ClustalW and analyzed with 11 reference sequences from GenBank. Figure 5 shows the phylogenetic tree of the 25 sequences with maximum likelihood analysis. All the 14 isolates were clustered in genotype II.

## Discussion

Guangzhou has an ideal environment for DENV transmission; it has a subtropical climate, a population of over 10 million, presence of the vector mosquito, and frequent interaction with countries that have DENV epidemics. In total, over 10,000 DENV infection cases and 9 major outbreaks (over 200 infection cases each time) have been reported in the last 34 years in China [14,16]. The statistical data from the Chinese Center for Disease Control and Prevention showed that from 2005 to 2010, there were 2198 reported DENV infection cases in China, and 42.63% (937 cases) of them were in Guangzhou alone, which is the highest among all cities. Given this statistic, we can say that the epidemiological and virological characteristics of DENV in Guangzhou are representative of the situation in China as a whole.

In Guangzhou, the rainy season extends from April to September, and the average temperature in Guangzhou between April and October is usually above 20°C [17]. Our study showed that the density of *Ae*. *albopictus* and its larvae reached the highest level between June and August in Guangzhou [18]. Those facts can explain why the DENV epidemic in Guangzhou peaks between August and October.

To determine which strains were prevalent in Guangzhou, we performed phylogenetic analysis on the full-length E gene sequenced from 90 DENV strains that had been isolated from clinical samples [19]. DENV-1 was predominant in most of the years between 2001 and 2010 (Table 2). It was also the only serotype detected every year between 2001 and 2008, except in 2005. However, in 2009, infections caused by DENV-1, 3, and 4 serotypes occurred, and the incidence of DENV-3 prevailed over DENV-1. Then, in 2010, all 4 serotypes appeared, and again, DENV-3 predominated. The phylogenetic tree of DENV-1 showed that genotype replacement occurred during this decade. The DENV-1 strain observed in 2001 was of genotype I; however, it was replaced by genotype IV in 2002, 2003 and 2004. In 2005, the dominant strain, again, was genotype I, and it remained predominant until 2010. In 2007 and 2010, both genotype I and genotype IV co-circulated. In 2009, a new genotype, genotype V occurred. Interestingly, the strains in the phylogenetic tree of DENV-2, 3, and 4 show less diversity. The DENV-2 and 4 strains belonged to only one genotype, whereas the DENV-3 strains were divided into two genotypes. There is little evidence to support the idea that there were genotype shifts in DENV-2, 3, and 4. It could be possible that they all just appeared from 2009 (except for DENV-2 that occurred in 2005). As we have demonstrated, a lengthy longitudinal study is required to observe these shifts.

After years of investigation, we found no serotype or genotype was established in Guangzhou. Among all the DENV infection cases reported in Guangzhou, 80 (3.23%) cases were imported. Imported cases were seen every year between 2001 and 2010. The sensitivity of the population may also be another reason. No death or DHF/DSS case was found in the 2478 DENV infection cases during this decade. This incidence is quite low compared with other Southeast Asia countries. The incidence rate of DHF/DSS in Malaysia, Singapore, and Vietnam was 4.07–8.5, 10.47, and 51.01–77.65 per 1 million people from 2001 to 2010, as indicated on the WHO website [20]. Research has shown that some human genes are associated with DENV infection severity [21]. It could be that the people in Guangzhou are not as sensitive to DENV as the residents from other countries. Further genetic and epidemiological research would be required to determine if this is the case.

Our epidemiological investigation showed that Southeast Asian countries were the main sources (86.25%) of the imported DENV cases (Table 2). The WHO reported that the Southeast Asian region together with the Western Pacific region bears nearly 75% of the current global dengue burden. At present, from these regions, only the Democratic People’s Republic of Korea has no reports of indigenous dengue cases [22]. Southeast Asian countries are the favored travel destination for people living in Guangzhou [23]. Most of these Southeast Asian countries have had severe DENV epidemics and all 4 serotypes have, at some point in time, been in circulation. The increased risk of DENV infection for travelers in Southeast Asia therefore increases the risk of transmission to local people in Guangzhou. The BLAST results and the phylogenetic trees of the isolated strains support this hypothesis. For the imported cases, the phylogenetic analysis verified our epidemiological data. For the local cases, most of the strains in Guangzhou were closely related to the strains isolated in prior epidemics in Southeast Asian countries. For DENV-1, the isolates in 2002 were close to the strains in Indonesia in 1998; those in 2007 were close to the strains in Malaysia in 2004; and those in 2008 were close to the strains in Vietnam in 2003. For 2010, the DENV-2 isolates were close to the strains in Vietnam in 2006, the DENV-3 isolates were close to the strains in Indonesia in 2007, and the DENV-4 isolates were close to the strains in Indonesia in 2004. A similar outcome has been reported by Wu *et al*. [24]. These results provide evidence to support the theory that Southeast Asian countries are the possible source of DENV infection in Guangzhou.

DENV-1 caused 2 major outbreaks in Guangzhou between 2001 and 2010, that is, one in 2002 (1423 cases) and another in 2006 (779 cases). Although they had the same serotype and similar impact, the strains causing the 2 outbreaks had different origins. The phylogenetic trees showed that the isolates from those two outbreaks had 2 different genotypes. The isolates in 2002 belonged to genotype IV and were close to the strain separated in Indonesia in 1998. At the beginning of the outbreak in Guangzhou in 2002 (May), a patient who came from Indonesia was found to have DENV infection. He was the first patient to be infected in 2002; however, viral isolation was unsuccessful in this case. Therefore, it cannot be concluded that the prevalent strain in 2002 came from Indonesia. Similarly, the isolates in 2006 belonged to genotype I. Cambodia was the most prevalent source of DENV infection cases in 2006. There were 8 import cases in Guangzhou that year, 4 (50%) of which came from Cambodia. They were detected in April, August, September, and October. This indicates that in 2006, DENV infection was detected in patients coming from Cambodia at the beginning and during the peak of the outbreak. This is similar to the situation that occurred in 2002, and again, no imported strain was isolated in 2006. Therefore, because of the absence of virus strains from these imported cases, and the lack of epidemiology information in Indonesia (2002) and Cambodia (2006), it’s insufficient to draw a conclusion. However, there may be some connection between the outbreaks and the imported cases.

The diversity in DENV genotypes and serotypes has increased in recent years; almost only DENV-1 was separated during 2001–2008, in contrast, the strains from 2010 included not only all 4 serotypes, but also different genotypes within the same serotype. Researchers believe that the introduction of a new serotype or genotype in an area brings with it a high possibility of a new epidemic. The high diversity of DENV in recent years indicates that there is a hidden risk of DENV epidemics occurring in Guangzhou in the near future, probably caused by the import of strains with new serotypes or genotypes.

## Conclusions

Our study suggested that August to October is the epidemic season of DENV. Strain and genotype shift were frequent during the last decade and no serotype or genotype was established in Guangzhou. DENV strains from different years had different origins. Southeast Asian countries were found to be the most likely source of DENV in Guangzhou. Along with the increasing numbers of imported DENV cases and the diversity of DENV strains, Guangzhou is at an increased risk of DENV outbreak in the near future.

## Methods

### Ethics statement

This study was approved by the Ethics Committee of the Guangzhou Center for Disease Control and Prevention. Written informed consent was obtained from all participants in the study.

### Viruses

The DENV strains used in this study were isolated from patients’ sera, which were collected by the Guangzhou Center for Disease Control and Prevention (Guangzhou CDC) from 2001 to 2010 in Guangzhou City. The serum samples were diluted 50 fold with RPMI-1640 (Gibco, USA) and then used to inoculate a C6/36 cell monolayer, which was then incubated at 28°C for 10–14d. The DENV positive cell culture was verified by indirect immunofluorescence(IIF) test. The culture supernatant was recovered by centrifugation and stored at −80°C.

### RNA extraction, RT-PCR, and sequencing

RNA was extracted from cell culture supernatants using QIAamp Viral RNA Mini kit (Qiagen, Germany), according to the manufacturer’s instructions. cDNA was synthesized by reverse transcription with SuperScript II Reverse Transcriptase (Invitrogen, USA). The procedure followed the manufacturer’s instruction. Two primers were designed to amplify the full length E gene (1.7 kb): DEN750 (5’-CAAGAACCGAAACGTGGATG-3’) and DEN2639 (5’-TGTGGAAGCAAATATCACCTG-3’). The PCR was performed using 0.5 μL of each primer and 4 μL of cDNA, which were added to 45 μL Platinum PCR SuperMix (Invitrogen, USA). The PCR reaction was initiated with a denaturation step at 94°C for 2 min; followed by 40 cycles of denaturation (94°C, 30 sec), primer annealing (52°C, 30 sec) and primer extension (68°C, 3 min); and ended with an extension step at 72°C for 7 min. Amplified products were purified by agarose gel electrophoresis and sequenced using ABI 3730 Genetic Analyzers (Applied Biosystems, USA).

### Phylogenetic analysis

Ninety sequences from isolated strains and 91 reference sequences from GenBank were aligned with ClustalW implemented in the MEGA software version 4.0 package. Phylogenetic trees were constructed with the maximum parsimony and maximum likelihood (ML) methods incorporated in the MEGA software version 4.0 package by using the Kimura 2-parameter model. The bootstrap value was 1000 replicates and only values over 50 are presented.

## Competing interests

The authors declare that they have no competing interests.

## Authors’ contributions

LJ carried out most of the experiments and wrote the material and methods section. XW designed the experiments and wrote most of the manuscript. YW, ZB, and YX performed virus cultures and gene sequencing. QJ, LL, and ZD collected the epidemiological information. YC, DB, and YW participated in DENV detection analysis. ZY and MW performed the data analysis. All authors read and approved the final manuscript.
